# The negative association between trait mindfulness and post‐traumatic stress disorder: A 4.5‐year prospective cohort study

**DOI:** 10.1002/brb3.2163

**Published:** 2021-08-06

**Authors:** Lionel Gibert, Wissam El Hage, Charles Verdonk, Bernard Levy, Bruno Falissard, Marion Trousselard

**Affiliations:** ^1^ IRBA: Institut de Recherche Biomédicale des Armées Brétigny sur Orge France; ^2^ INSERM: Institut National de la Santé et de la Recherche Médicale Paris France; ^3^ Centre Hospitalier Universitaire Paul Brousse Unité de Recherche Psychiatrie‐Comorbidités‐Addictions PSYCOMADD Villejuif France; ^4^ Centre Hospitalier Universitaire de Tours Tours France

**Keywords:** aggression, early intervention, epidemiology, PTSD, risk factors

## Abstract

**Objective:**

Post‐traumatic stress disorder (PTSD) is a chronic, disabling condition. Our main objective is to investigate the association between trait mindfulness and PTSD over a period of 54 months. The secondary objective is to provide an exhaustive description of PTSD trajectories after the Bataclan attack.

**Methods:**

We designed a prospective cohort study of 133 subjects present in the Bataclan concert hall during the November 2015 terrorist attack in Paris, France. Data were recorded 6, 18, 30, and 54 months after the attack. The primary endpoint was evaluated using the PTSD Check List Scale. Trait mindfulness was measured by the 14‐item Freiburg Mindfulness Inventory.

**Results:**

FMI scores were consistently, significantly, and negatively associated with PCL‐5 scores. Adjusted odds ratios were at 0.81 (6 months), 0.88 (18 months) 0.82 (30 months), and 0.81 (54 months). PTSD prevalence 6 months after the event was 77%; it remained at 41% after 54 months. PTSD status of subjects is fluctuating. Latent class analysis divided the cohort into 3 groups: 21% of subject who remained below PTSD threshold throughout, 30% who remained above throughout, and 49% who steadily reduced their PTSD scores over time.

**Conclusion:**

In our cohort, mindfulness is negatively associated with PTSD. Mindfulness programs are designed to improve global resilience and treat anxiety and mood disorders. Further research is needed to investigate if improving trait mindfulness is possible and beneficial for patients suffering from PTSD.

## Graphical abstract

PTSD prevalence remains very high for direct victims of terrorism even after 54 months. We found a strong negative association between trait mindfulness and post‐traumatic stress disorder at 6, 18, 30, and 54 months. Mindfulness prevention programs for post‐traumatic stress disorder should be implemented as soon as possible after the trauma.

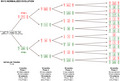

## 1 SIGNIFICANT OUTCOMES

‐ We found a strong negative association between trait mindfulness and post‐traumatic stress disorder at each phase of the study (6, 18, 30, and 54 months).
‐ PTSD prevalence remains very high for direct victims of terrorism even after 54 months.

## 2 LIMITATIONS

‐ Our cohort is relatively small (133 subjects) even if it represents about 8% of survivors to the attacks.

## 3 CLINICAL RECOMMENDATIONS

‐ Mindfulness prevention programs for post‐traumatic stress disorder should be implemented as soon as possible after the trauma.

## 4 INTRODUCTION

Post‐traumatic stress disorder (PTSD) is a chronic, disabling condition (Yehuda et al., 2015). Six months after a traumatic event, prevalence among victims of terrorist attacks has been found to be over 75% (Gibert et al., 2018). It is associated with substance abuse (Berenz et al., 2017), mood and anxiety disorders (Knowles et al., 2019), suicide (Shen et al., 2016), and somatic problems (Nichter et al., 2019). As there are few therapeutic options, and the outcome is often uncertain (Shalev et al., 2017), measures need to be taken to prevent this public health issue (Qi et al., 2016). Several psychological approaches, based on primary interventions, have been tested. However, to the best of our knowledge, very few are based on mindfulness (Roberts et al., 2019). At the same time, no pharmacological interventions have proven to be efficient in preventing PTSD in the early stages after a trauma (Amos et al., 2014). PTSD symptomatology tends to fluctuate over time (Solomon, 2006), and secondary prevention is typically limited to monitoring symptoms.

On 13 November 2015, terrorists broke into the Bataclan concert hall in Paris, France, armed with automatic weapons. Among the 1,500 spectators, 130 were killed, and 450 injured. In our earlier study, run 6 months after the event, we found a strong negative association between PTSD and trait mindfulness (Gibert et al., 2018). We continued our work, as we believed that analyses of long‐term trajectories would help to consolidate these preliminary results, and better‐understand the impact of trait mindfulness on the evolution of PTSD. Our approach is all the more important, as trait mindfulness can be modified with long‐term, regular training (Lang et al., 2012), while meditation practice increases attentional and emotional control, and encourages acceptance of the situation (Tang et al., 2015).

### 4.1 Aims of the study

The main objective of this study is, therefore, to investigate the association between trait mindfulness and PTSD over a period of four and a half years. The secondary objective is to provide an exhaustive description of PTSD trajectories after the Bataclan attack.

## 5 METHOD

### 5.1 Participants

**FIGURE 1:**
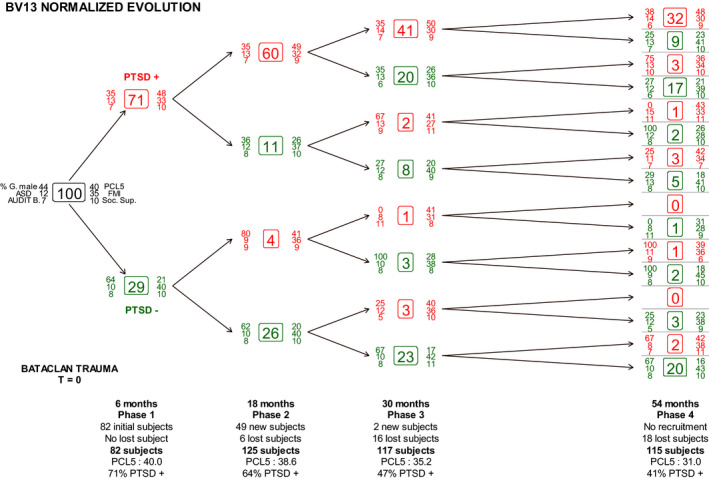
Post‐traumatic stress disorder (PTSD) trajectories. PCL‐5: PTSD checklist; FMI: the Freiburg Mindfulness Inventory; AUDIT: Alcohol Use Disorders Identification Test; ASD: Acute Stress Disorder; Soc. Sup.: social support; PTSD + indicates subjects with a PCL‐5 score of 33 or over

Subjects were recruited through the association “Life for Paris,” which was setup for victims of the attack. The main inclusion criterion was being present in the Bataclan concert hall on the night of the event. About 8% of survivors participated in the study. Victims were enrolled between 12 April 2016, and 5 August 2018 (Figure 1).

### 5.2 Procedure

The study was conducted in accordance with all applicable regulatory requirements and approved by the Ethics Committee at Tours University Hospital (ClinicalTrials.gov identifier NCT02853513). All volunteers provided written, informed consent before participation. Our project received moral and financial support from the French National Center for Scientific Research (CNRS “Attentats‐Recherche”).

### 5.3 Measures

Data were recorded at 6, 18, 30, and 54 months after the attack, through self‐assessment questionnaires administered via the internet.

The primary endpoint was evaluated using the PTSD Check List Scale (PCL‐5) (Ashbaugh et al., 2016), which is in line with the DSM‐5 definition of PTSD, at a threshold score of 33 (American Psychiatric Association, 2013). Trait mindfulness was measured by the 14‐item Freiburg Mindfulness Inventory (FMI). A score over 38 indicated an efficient mindful functioning (Trousselard et al., 2010; Walach et al., 2006). Social support was assessed by a Likert scale that measured perceived support from family, friends, and professionals. Socio‐demographic characteristics were also collected. Three items focused on medical history (past psychological treatment, trauma), and current mental health treatments in the form of simple (yes/no) closed questions. In phase 4 (at 54 months), subjects were invited to answer an open, optional question describing their health path.

Five items examined the state of the subject during the terrorist attack: alcohol consumption (number of drinks); duration of exposure to danger; automatic firearm injury; seeing the terrorists; and the loss of a loved one.

Acute stress disorder (ASD) was assessed by six items summarizing the following DSM‐5 criteria: negative mood; intrusion; avoidance; dissociative flashbacks; insomnia; and arousal. Peritraumatic dissociation was measured by the 10‐item Peritraumatic Dissociative Experiences Questionnaire Self‐Report Version(PDEQ‐SER) (Birmes, Carreras, Ducassé, et al., 2001); A score greater than 22 indicated a clinically relevant peritraumatic dissociation (Birmes, Carreras, Charlet, et al., 2001; Birmes et al., 2005). We evaluated alcohol use with the French validated Alcohol Use Disorders Identification Test (AUDIT) (Gache et al., 2005) at the time of the event and at the time the questionnaires were administered. The AUDIT test is an adapted screening instrument to measure alcohol consumption independently from the presence of dependence or of an AUD. We adjusted scores between males and females as recommended adding one point to female scores. Total scores of 8 or more are recommended as indicators of hazardous and harmful alcohol use (Babor et al.). AUDIT scores were not collected in phase 1 questionnaires. ASD and PDEQ data were not collected in phase 4, as there was no new recruitment.

### 5.4 Data analysis

All statistical analyses were performed with R software (v. 3.6.3). The description of the population was complemented by a flow chart showing PTSD trajectories (Figure 1). Latent class analysis was run with the PoLCA R package. Categorical explanatory variables were analyzed with a univariate ANOVA, and Pearson's correlation coefficient was used for quantitative variables. Variables with *p* values below 0.10 in the univariate model were used to build a multivariate logistic regression with PTSD status as the dependent variable and FMI as the explanatory variable. We also calculated unadjusted odds ratios for FMI scores. The significance level was set at *p* ≤ .05. We applied the same analysis in all four phases of the study. No participant was excluded from any of the analyses.

## 6 RESULTS

### 6.1 Population

The cohort consisted of 133 subjects. Eighteen were lost in the last phase (54 months after the attack). Sixty‐six answered in all for phases and one hundred and nine in phases 2, 3, and 4. (Figure 1). In our cohort, women were slightly over‐represented, and two‐thirds of the population was below 35 years old. No particular family situation predominated. Most subjects were highly educated, and graduates were over‐represented (Table 1).

**TABLE 1:** Categorical explanatory variables: description and univariate analysis (ANOVA)

	6 months					18 months					30 months					54 months				
	*N*	%	PCL−5	*SD*	*p*	*N*	%	PCL−5	*SD*	*p*	*N*	%	PCL−5	*SD*	*p*	*N*	%	PCL−5	*SD*	*p*
	82		40.1	15.4		125		38.6	16.3		117		35.2	16.3		115		31.0	16.1	
Demography																				
Female	47	57%	43.0	14.0	.05	71	57%	41.7	14.8	.01	67	57%	36.9	16.5	.18	69	60%	32.0	15.8	.39
Male	35	43%	36.0	16.4		54	43%	34.5	17.3		50	43%	32.8	16.0		46	40%	29.4	16.7	
18–35 years old	50	61%	41.7	14.7	.23	69	55%	40.1	15.6	.25	51	44%	36.0	15.0	.62	54	47%	30.2	14.8	.64
Over 35 years old	32	39%	37.4	16.2		56	45%	36.7	17.1		66	56%	34.5	17.4		61	53%	31.6	17.2	
Single, no children	25	30%	38.4	12.8	.45	40	32%	40.4	13.9	.56	33	28%	37.1	14.9	.69	35	30%	31.4	16.0	.26
Couple without children	26	32%	44.0	15.6		42	34%	39.7	17.1		39	33%	34.8	16.7		39	34%	28.3	14.3	
Couple with children	25	30%	37.5	18.0		34	27%	35.4	17.8		36	31%	35.1	17.5		34	30%	34.9	18.4	
Other	6	7%	40.3	11.6		9	7%	37.3	16.8		9	8%	29.7	15.7		7	6%	25.0	11.4	
High school	21	26%	47.3	12.2	.01	44	35%	41.5	16.6	.15	42	36%	37.0	17.8	.39	38	33%	32.6	16.7	.46
Graduate	61	74%	37.6	15.6		81	65%	37.0	16.0		75	64%	34.2	15.5		77	67%	30.2	15.9	
Nonmanagerial	22	27%	40.0	15.3	.94	28	22%	40.3	15.9	.49	24	21%	35.9	14.6	.17	21	18%	31.1	12.4	.41
Manager	33	40%	41.0	17.0		42	34%	37.1	18.1		39	33%	34.9	19.2		38	33%	32.7	18.1	
Independent	10	12%	35.8	15.3		12	10%	31.4	16.5		14	12%	26.7	10.7		13	11%	23.0	12.0	
Student	3	4%	45.7	5.1		7	6%	45.1	10.0		3	3%	49.3	7.5		3	3%	41.0	16.8	
Unemployed	4	5%	38.5	12.9		20	16%	40.7	16.0		17	15%	34.0	13.1		17	15%	29.5	16.0	
Other	10	12%	40.4	14.7		16	13%	39.6	14.1		20	17%	39.7	17.3		23	20%	32.4	19.9	
Peritraumatic Exp																				
0–30 min. exp.	32	39%	37.9	14.1	.31	53	42%	39.2	17.8	.75	46	39%	33.5	17.2	.38	43	37%	30.1	16.5	.65
> 60 min. exp.	50	61%	41.4	16.1		72	58%	38.2	15.2		71	61%	36.3	15.7		72	63%	31.5	16.0	
Saw terrorist: no	26	32%	40.2	16.6	.94	39	31%	40.1	14.5	.48	41	35%	36.6	17.3	.48	37	32%	31.1	17.6	.97
Saw terrorist: yes	56	68%	40.0	14.9		86	69%	37.9	17.0		76	65%	34.4	15.8		78	68%	30.9	15.5	
Loss of a loved one: no	70	85%	39.9	15.3	.79	110	88%	38.5	16.6	.89	103	88%	35.4	16.8	.70	102	89%	31.6	16.5	.24
Loss of a loved one: yes	12	15%	41.2	16.2		15	12%	39.1	13.7		14	12%	33.6	12.9		13	11%	26.0	11.7	
Wounded: no	68	83%	40.0	15.9	.97	105	84%	38.5	16.5	.83	100	85%	34.5	16.5	.29	96	83%	31.4	16.0	.56
Wounded: yes	14	17%	40.2	13.0		20	16%	39.3	15.3		17	15%	39.1	15.4		19	17%	29.0	16.9	
1 drink or less	56	68%	40.9	15.5	.49	79	63%	39.8	16.6	.27	79	68%	36.5	16.6	.20	77	67%	33.0	15.3	.07
More than 1 drink	26	32%	38.3	15.3		46	37%	36.5	15.6		38	32%	32.4	15.5		38	33%	26.9	17.0	
History																				
Trauma history: no	60	73%	38.1	15.5	.06	95	76%	37.9	16.5	.38	86	74%	35.0	15.3	.82	84	73%	30.7	15.5	.79
Trauma history: yes	22	27%	45.4	14.0		30	24%	40.9	15.7		31	26%	35.7	19.1		31	27%	31.6	17.8	
Psychiatric history: no	59	72%	39.1	16.5	.35	94	75%	38.5	16.3	.94	82	70%	34.5	15.9	.52	83	72%	31.2	16.4	.84
Psychiatric history: yes	23	28%	42.6	11.8		31	25%	38.8	16.3		35	30%	36.7	17.5		32	28%	30.5	15.4	
Treatment																				
No treatment	16	20%	31.8	14.9	.02	24	19%	33.0	18.9	.06	28	24%	30.2	16.4	.06	28	24%	27.4	15.8	.18
Treatment	66	80%	42.0	15.0		101	81%	39.9	15.4		89	76%	36.8	16.1		87	76%	32.1	16.1	

Note

Shaded boxes: significant results.

### 6.2 PTSD

PTSD prevalence was very high (77%) six months after the event. Although average PCL‐5 scores consistently fell by 2.25 points per year, they remained just below pathological level. After 54 months, 41% of subjects still suffered from PTSD. This can be compared to 32% of subjects who suffered from PTSD at each phase of the study, and 20% of subjects who did not suffer from PTSD at any time (Figure 1). The latent class analysis similarly divided the cohort into 3 groups: 21% of subject who remained below the PTSD threshold throughout, 30% who remained above it throughout, and 49% who steadily reduced their PTSD scores over time. Access to treatment was excellent. Among subjects with PTSD after six months, 95.1% received mental health treatment, and this proportion reached 98.3% 54 months after the event.

### 6.3 Situation during the attack

More than half of subjects remained in the building throughout the attack. Two‐thirds saw a terrorist, and 15% were wounded by gunfire (Table 1). Most suffered from ASD, with varying intensity. The average PDEQ score was above the usual threshold of 22, indicating clinically relevant dissociation (Birmes, Carreras, Charlet, et al., 2001; Birmes et al., 2005). When asked to describe their health path (at 54 months), 102 of the cohort of 117 answered the question. Only three noted mindfulness‐related treatment.

The univariate analysis showed that among the categorical explanatory variables (Table 1), none were significant in all four phases. Treatment was significant in the first three phases and gender in the first two. It also showed that for quantitative variables (Table 2), FMI, PDEQ, and ASD scores were significantly correlated to PCL5 scores in all four phases, while AUDIT before was in the last three phases. The multivariate analysis (Table 3) highlighted that odds ratios for adjusted and unadjusted FMI scores were the only consistently significant result in all four phases. We did the analysis and found the same results on the sixty‐six subjects that participated in all four phases. Furthermore, a consistent, strong negative association was identified between PCL‐5 and FMI scores (Table 2).

**TABLE 2:** Quantitative explanatory variables

	Average	*SD*	Pearson's r	*p*
6 months				
FMI	35.5	6.8	−0.54	<.001
ASD score	12.0	4.0	0.59	<.001
PDEQ	23.5	8.6	0.19	.08
Social support	9.9	1.5	−0.18	.10
AUDIT before	NA	NA	NA	NA
Present AUDIT	NA	NA	NA	NA
18 months				
FMI	34.3	7.8	−0.53	.10
ASD score	12.6	4.2	0.50	<.001
PDEQ	26.5	9.7	0.35	<.001
Social support	9.4	1.6	−0.34	<.001
AUDIT before	7.0	4.4	−0.12	.20
Present AUDIT	8.8	6.8	0.09	.32
30 months				
FMI	34.6	7.7	−0.67	<.001
ASD score	13.0	3.9	0.52	<.001
PDEQ	26.3	10.1	0.27	.03
Social support	9.4	1.6	−0.42	<.001
AUDIT before	6.7	4.0	−0.21	.02
Present AUDIT	8.2	6.4	0.11	.24
54 months				
FMI	36.7	8.2	−0.64	<.001
ASD score	12.9	3.9	0.35	<.001
PDEQ	25.5	9.9	0.18	.06
Social support	9.4	1.8	−0.34	<.001
AUDIT before	6.8	4.0	−0.32	.001
Present AUDIT	8.1	6.1	0.12	.22

Note

Mean, standard deviation and Pearson's correlation with PCL‐5 score.

Abbreviations: ASD: Acute Stress Disorder; AUDIT: Alcohol Use Disorders Identification Test. Shaded boxes: significant results;FMI: Freiburg Mindfulness Inventory; PDEQ: Peritraumatic Dissociative Experiences Questionnaire.

**TABLE 3:** Results of the multivariate logistic regression analysis

	OR	CI 95		*p*
6 months				
Unadjusted FMI	0.83	0.75	0.91	<.001
Adjusted FMI	0.81	0.70	0.91	<.001
Gender	0.18	0.03	0.77	<.05
ASD score	1.24	1.03	1.54	<.05
PDEQ	1.08	0.98	1.21	.15
Treatment	0.40	0.06	2.24	.3
Social Support	0.90	0.50	1.55	.7
Trauma history	1.19	0.20	8.25	.8
Education	0.87	0.12	5.52	.9
18 months				
Unadjusted FMI	0.86	0.81	0.92	<.001
Adjusted FMI	0.88	0.82	0.94	<.001
PDEQ	1.04	0.99	1.10	.09
ASD score	1.09	0.97	1.22	.17
Social Support	0.82	0.60	1.08	.17
Treatment	1.45	0.47	4.46	.51
Gender	0.82	0.34	2.00	.67
30 months				
Unadjusted FMI	0.83	0.76	0.89	<.001
Adjusted FMI	0.82	0.74	0.90	<.001
PDEQ	1.08	1.02	1.14	<.01
AUDIT before	0.85	0.73	0.97	<.05
ASD score	1.11	0.95	1.31	.20
Treatment	1.72	0.51	5.95	.4
Social Support	0.95	0.69	1.32	.8
54 months				
Unadjusted FMI	0.81	0.74	0.87	<.001
Adjusted FMI	0.81	0.73	0.88	<.001
Social Support	0.73	0.54	0.95	<.05
AUDIT before	0.87	0.75	1.00	.07
Alcohol consumption	3.0	0.49	19.13	.229
PDEQ	1.03	0.97	1.08	.33
ASD score	1.01	0.89	1.16	.86

Note

Abbreviations: ASD: Acute Stress Disorder. Shaded boxes: significant results; AUDIT: Alcohol Use Disorders Identification Test; CI: Confidence Interval. FMI: Freiburg Mindfulness Inventory; OR: Odds ratio; PDEQ: Peritraumatic Dissociative Experiences Questionnaire.

## 7 DISCUSSION

Our main results show a strong, negative association between PCL‐5 and FMI scores in all phases of the study—from six months to 4.5 years (54 months). Assuming these scores correctly represent PCL‐5 symptoms and trait mindfulness, we can confirm that PTSD and mindfulness are strongly associated. As for the secondary objective, our cohort study confirms the change in PTSD status as time passes (Figure 1). Similar trends have been observed for military veterans (Solomon, 2006). Primary and secondary interventions are, therefore, of paramount importance.

Among the variables significantly associated with PTSD in our multivariate model (Table 3), we can distinguish two groups. The first contains those that cannot be changed: gender, pretrauma AUDIT, ASD symptoms, and PDEQ scores. The second contains those that are modifiable: FMI and social support. The first group helps physicians, in the very early stages after the trauma, to identify those patients who are most at risk. This is all the more crucial as the influx of victims can be massive. However, after six months, the best indicator of PTSD is the PCL‐5 itself, and the first group of risk factors loses its clinical relevance. The second group of variables are more useful tools in primary and secondary prevention.

Gender (female) is associated with PTSD in our cohort, as classically found in the literature, but only in phase 1 (Shalev et al., 2019). Gender differences in stress‐induced alterations in cognition, arousal, and fear response could explain this result (Bangasser & Wicks, 2017). It might also be the case that women are more at risk of ASD, for a period of over one month.

Pretrauma AUDIT and PCL‐5 scores were associated in phases 3 and 4. The first explanation could be that alcohol consumption on the evening of the event could have altered memory mechanisms and be protective (Maes et al., 2001). However, this did not appear to be the case among our cohort. The second hypothesis is that alcohol is efficient in diminishing PTSD symptoms. For obvious reasons, this result cannot be used in prevention programs; high scores are not protective in the long term, as chronic alcohol consumption can increase anxiety (Becker, 2017). Links between PTSD and alcohol use disorder are complex (Berenz et al., 2017). 24.2% of individuals with lifetime PTSD meet criteria for lifetime alcohol dependence, compared to 13.7% of those without a history of PTSD (National Epidemiological Survey on Alcohol and Related Conditions) (Blanco et al., 2013).

ASD was associated with the development of PTSD, but only six months after the attack. The lack of continuity between ASD and PTSD confirms the distinction between the two disorders. The DSM‐5 classification puts the tipping point between ASD and PTSD at one month. Our results suggest a point somewhere between 6 and 18 months.

The positive association between PCL‐5 and PDEQ seems to be stronger than the one with ASD. We found this positive association in phase 3, along with a tendency in phase 2, consistent with the literature (Birmes et al., 2003).

Two modifiable variables were associated with PCL‐5 scores: social support and mindfulness. This observation confirmed our initial results, based on data collected six months after the attack.

As expected, perceived social support is negatively associated with PCL‐5 in our cohort, but only 54 months after the event. Although we found that it had little impact at the onset of PTSD, early caring has shown to be somewhat efficient in other studies (Roberts et al., 2019). Some authors have argued that a perceived lack of social support only predicts depression, and not PTSD (Adams et al., 2019). On the one hand, social functioning increases resilience in the face of trauma, and on the other hand, PTSD leads to social dysfunction (Stevens & Jovanovic, 2019). Further investigation is needed to understand the links between perceived social support, real social support, social cognition, and maybe even attachment personality. Prevention programs could, nevertheless, seek to improve the perception of social support among those suffering from PTSD.

### 7.1 Mindfulness

Our main result is the strong and stable, negative association between trait mindfulness and PTSD, respectively, characterized by FMI and PCL‐5 scores (Table 3). Although the gold standard for diagnosing PTSD is the Clinician‐Administered PTSD Scale (CAPS‐5), the PCL‐5 is widely accepted as a very good proxy (Blevins et al., 2015). There is no gold standard for trait mindfulness but the FMI 14‐item scale is considered very robust (Walach et al., 2006). We chose this instrument as it has been designed to measure core trait mindfulness in subjects who do, or do not, meditate.

“Paying attention in a particular way: on purpose, in the present moment, and nonjudgmentally” is widely accepted as a definition of mindfulness (Kabat‐Zinn, 1990). Physical sensations are observed and accepted, as they come. Attentional anchors, such as respiration, help to bring the focus back to the base. Mindfulness training has been shown to reduce arousal, improve emotion control, and acceptance of unwanted experiences (Keng et al., 2011). These issues address the core PTSD symptoms (anxiety, arousal, avoidance). Mindfulness programs have been shown to be efficient in treating PTSD symptoms (Polusny et al., 2015).

Although there are inter‐individual differences in the propensity to be mindful, trait mindfulness can be modified by regular training (Brown & Ryan, 2003; Kiken et al., 2015). According to Johnson et al., “*Mindfulness training in Marines preparing for deployment showed that mechanisms related to stress recovery can be modified in healthy individuals prior to stress exposure*” (Johnson et al., 2014). Mindfulness practice is thought to impact connectivity within the parts of the brain involved in pathological PTSD processes. The hippocampus (memory processes), and the anterior cingulate cortex, mid‐cingulate cortex, and orbitofrontal cortex (self and emotion regulation) have been found to be impacted by mindfulness training (Tang et al., 2015). Mindfulness programs have been used and proven to be useful in the prevention of a variety of psychiatric conditions: addiction problems (Shonin & Van Gordon, 2016), anxiety disorders, and stress reduction (Janssen et al., 2018), and to avoid depression relapse (Kuyken et al., 2016).

The mechanisms of mindfulness can be studied by separating the two main pillars of the practice: paying attention to the present and acceptance. Attention training has been shown to have an impact on PTSD symptoms among Israeli and United States combat veterans (Badura‐Brack et al., 2015). On the other hand, acceptance skills are associated with fewer depressive, anxiety, and stress symptoms (Boden et al., 2012). In our view, acceptance cannot be trained independently of attention monitoring, while attention without acceptance is not mindfulness. The Monitor and Acceptance Theory (MAT) paradigm integrates the two notions in the description of mindfulness training (Lindsay & Creswell, 2017). In this paradigm, monitoring and acceptation interact to produce health benefits. Being present, without acceptance, would not produce the same effects, as a negative experience could worsen symptoms (Lindsay & Creswell, 2019). Mindfulness‐Based Stress Reduction is an established program that integrates both attention monitoring and acceptance. It has been found to have a positive impact on anxiety and mood disorder, independent of diagnosis (Khoury et al., 2015), and could, therefore, be a useful tool in primary and secondary prevention.

Recruitment was done through an association of victims (Life For Paris), which can create a bias. Associations are usually joined before the onset of PTSD and are more related to the status of victim than to a medical condition. The main reasons for joining are to share experiences and to benefit from support for administrative work, as applying for financial compensation is a rather laborious process. If severe symptoms may have prevented some subjects from completing the questionnaires, it can be assumed that subjects without symptoms did not feel the need to join an association. We cannot calculate the exact impact of these biases. Our cohort represents 8% of those who survived the attack (approximately 50% are members of the association Life For Paris) and therefore should still be representative of all spectators at the Bataclan.

A limitation of our study is that we did not investigate the nature of the treatment received by subjects in our cohort. Receiving treatment (or not) was not significantly associated with PCL‐5 scores in any phase, although a large majority of subjects benefitted from professional care. In the open, optional question about their health path, words related to mindfulness only appeared three times, which is coherent with the stable FMI scores reported by our cohort. We therefore conclude that this limitation did not affect our main result.

We purposely chose to use cross‐sectional analyses because we decided ex‐ante to look for an association between PCL‐5 and FMI and its stability over time. We examined the longitudinal data and found that mindful subjects could deteriorate (not significantly) because their initial PTSD scores were very low.

Another limitation, which is inherent in any cohort study, is that our population changed slightly from one phase to another. We actively recruited more subjects after our preliminary results, and the phase 1 population is necessarily smaller. On the other hand, phase 2, 3, and 4 populations are quite similar.

A final limitation of our study is memory bias, in the investigation of ante‐traumatic or peritraumatic characteristics (ASD, PDEQ, and AUDIT scores). This is a structural issue in this type of cohort, as facing a trauma cannot be scheduled. To the best of our knowledge, no research has specifically examined memory bias in a long‐term, follow‐up study of traumatized subjects. Table 2 shows averages for each of the scores. It is difficult to compare phase 1 averages with the other phases as the cohort was smaller. ASD, PDEQ, and AUDIT scores appear to be stable over time in phases 2, 3, and 4. If there is any memory bias, it appears to be constant, although traumatic memories are classically inconsistent over time (Hepp et al., 2006). We conclude that this bias does not affect our main result.

Mindfulness is the clinical variable most closely associated with PTSD in our cohort. Mindfulness programs, first described in the early 1990s by Jon Kabat‐Zinn, have been implemented to improve global resilience and to treat anxiety and mood disorders. We found a strong negative association between trait mindfulness and PTSD. Further research is needed to investigate how improved trait mindfulness might benefit patients with PTSD.

## CONFLICT OF INTEREST

The authors have no conflict of interest to declare.

## AUTHOR CONTRIBUTION

LG, MT, and BF conceived and designed the analysis. LG collected the data. LG performed the data analysis under BF supervision. LG, MT, and CV wrote the first manuscript. WEH and BL reviewed the scientific part of the manuscript. All authors contributed to and have approved the final submitted version of the manuscript.

### PEER REVIEW

The peer review history for this article is available at https://publons.com/publon/10.1002/brb3.2163.

## Data Availability

The data that support the findings of this study are available from the corresponding author upon request.
